# Catheter-Related Late Complications in Cancer Patients During and After the COVID-19 Pandemic: A Retrospective Study

**DOI:** 10.3390/cancers17071182

**Published:** 2025-03-31

**Authors:** Alessio Lo Cascio, Mattia Bozzetti, Daniele Napolitano, Marcella Dabbene, Leonardo Lunetto, Roberto Latina, Stefano Mancin, Marco Sguanci, Michela Piredda

**Affiliations:** 1 La Maddalena Cancer Center, Via San Lorenzo 312, 90146 Palermo, Italy; alessio.locascio@hotmail.it (A.L.C.); dabbene.marcella@lamaddalenanet.it (M.D.); lunetto.leonardo@lamaddalenanet.it (L.L.); 2 Azienda Socio Sanitaria Territoriale di Cremona, 26100 Cremona, Italy; mattia.bozzetti@asst-cremona.it; 3 Cemad–Fondazione Policlinico Gemelli IRCCS, Largo Agostino Gemelli, 8, 00168 Rome, Italy; 4 Department of Health Promotion, Mother and Child Care, Internal Medicine and Medical Specialties, University of Palermo, Piazza delle Cliniche, 2, 90127 Palermo, Italy; roberto.latina@unipa.it; 5 IRCCS Humanitas Research Hospital, Rozzano, 20089 Milan, Italy; 6 Research Unit Nursing Science, Department of Medicine and Surgery, Campus Bio-Medico di Roma University, via Alvaro del Portillo, 21, 00128 Rome, Italy; sguancim@gmail.com (M.S.); m.piredda@unicampus.it (M.P.)

**Keywords:** catheter-related complications, COVID-19, PICC, midline, neoplasms, nursing

## Abstract

Background: Peripherally Inserted Central Catheters (PICCs) and midline catheters are crucial for chemotherapy and supportive care in cancer patients. Their use requires ongoing monitoring to prevent late complications such as infections, dislodgements, and replacements. The COVID-19 pandemic challenged healthcare systems, potentially increasing these complications due to reduced outpatient services and limited specialized personnel. Objectives: This study compared the incidence of late complications associated with PICCs and midline catheters in cancer patients during and after the COVID-19 pandemic. Methods: A retrospective observational study was conducted at a Cancer Center in Italy from March 2020 to April 2024. Catheter-related complications were divided into two cohorts: during the pandemic (March 2020–March 2022) and post-pandemic (April 2022–April 2024). The primary outcome was the incidence of late complications requiring device removal, categorized as infections, dislodgements, and replacements. Statistical analyses included the Chi-squared test for categorical variables and the Kruskal–Wallis test for continuous variables. Results: Of 4104 PICC and midline catheter placements, 2291 removals were recorded, with 550 (24%) due to late complications—404 during the pandemic and 146 post-pandemic (*p* < 0.001). Suspected infections were the most frequent complication, significantly higher during the pandemic (*p* < 0.001). Dislodgements and replacements also decreased markedly post-pandemic. Limited outpatient services and disrupted healthcare workflows likely contributed to higher complication rates during the pandemic. Conclusions: The COVID-19 pandemic negatively impacted catheter management in cancer patients, increasing late complications. The post-pandemic decline highlights the importance of consistent care, infection prevention, remote monitoring, and stronger healthcare resilience to reduce risks in future crises.

## Simple Summary

Cancer patients frequently require specific types of catheters, such as peripherally inserted central catheters (PICCs) and midline catheters, to facilitate the administration of chemotherapy and other treatments. However, these devices can lead to complications like infections, accidental dislodgement, or the need for replacement. During the COVID-19 pandemic, hospitals faced challenges such as fewer outpatient services and reduced specialized staff, which may have increased these complications. This study examined how often these problems occurred in cancer patients during and after the pandemic. The results showed that catheter-related complications were more frequent during the pandemic, especially infections. Afterward, complications significantly decreased, highlighting the importance of proper catheter management and continuous care. These findings underline the necessity of robust healthcare systems, infection prevention strategies, and remote monitoring to ensure safer treatment for cancer patients, including during future health crises.

## 1. Introduction

The use of Peripherally Inserted Central Catheters (PICCs) and midline catheters has become a cornerstone in the delivery of chemotherapy and supportive care for cancer patients [1,2]. These devices provide a reliable method of venous access, essential for administering prolonged treatments both within hospital and home settings [3,4]. Despite the clinical advantages of these devices, their management requires continuous monitoring to prevent late device-related complications, such as infections, dislodgements, and replacements [5,6,7]. These complications can result in treatment interruptions, increased morbidity, higher healthcare costs, and significant risks to patient safety [8,9].

Reported overall incidence rates for these complications are 15.9% for infections, 34% for thrombosis, and 40.7% for mechanical complications [10,11,12]. The rate of PICC-related bloodstream infections (BSIs) has been estimated at 2.1 per 1000 catheter days in hospitalized patients and 1.0 per 1000 catheter days in outpatient settings [13,14]. Recent investigations indicate that PICCs may be associated with a lower incidence of BSIs when compared to other types of central venous catheters (CVCs) [15,16,17]. However, the findings by Chopra et al. (2013) [18] suggest that when infection rates are standardized per catheter day, the incidence of BSIs associated with PICCs is comparable to that of CVCs.

Such variability in reported outcomes may be attributable to differences in patient populations (e.g., oncology) and types of infused therapies, including parenteral nutrition or long-term antibiotic administration. Additionally, the healthcare setting itself appears to be a significant determinant of PICC-related complication rates [16,18,19]

The onset of the COVID-19 pandemic in early 2020 posed unprecedented challenges to healthcare systems globally [20,21]. Cancer patients faced heightened risks during this period due to their condition of immunosuppression that makes them particularly vulnerable to infections [22]. The imperative for stringent infection control measures, coupled with the redirection of healthcare resources towards managing COVID-19 cases, severely disrupted routine medical services [23,24]. Limited access to outpatient care and reduced availability of specialized personnel for device management likely exacerbated catheter-related complications [25,26]. While previous research has explored various aspects of healthcare disruptions caused by the pandemic, studies specifically addressing complications related to venous access devices in cancer care are lacking [27,28]. This is critical as empirical data on the impact of the COVID-19 pandemic on cancer patients are essential to inform the development of robust strategies aimed at improving patient outcomes and ensuring continuity of care during future health emergencies.

However, despite the attention paid to the impact that COVID-19 has had on a global level, there are gaps in the correlation between this period and complications related to these devices.

This study seeks (1) to describe the incidence of late complications associated with PICCs and midline catheters in cancer patients; (2) to provide a comparative analysis of such complications during and after the COVID-19 pandemic period; and (3) to describe the patient characteristics associated with such complications.

## 2. Materials and Methods

### 2.1. Ethics Statement

The study was approved by the Research Ethics Committee of Palermo (Protocol number PICC 00Vr1.30.05.2023 n°06/2023).

### 2.2. Study Design

This retrospective observational study was conducted at a Cancer Center in Italy to compare the incidence of late complications associated with Peripherally Inserted Central Catheters (PICCs) and midline catheters in cancer patients during the COVID-19 pandemic and the post-pandemic period. Data were collected retrospectively from medical records, capturing patients’ demographic details including age and gender, cancer types, presence of metastases, comorbidities, and information on catheter placements and removals. The outcomes were the incidence of late complications necessitating device removal, categorized as infections, dislodgements, or replacements, the comparison between late complications occurring during and after the COVID-19 pandemic, and the patient and device characteristics associated with complications.

### 2.3. Current Strategies for These Complications’ Prevention

All devices were inserted under ultrasound guidance, with placement confirmed via intracavitary ECG, and the procedures were conducted in a protected clinical environment. The catheters used in the study were made of medical-grade polyurethane, a material chosen for its excellent biocompatibility, mechanical strength, and flexibility. This composition allowed for optimal hemodynamic performance, supporting high flow rates while ensuring patient comfort and minimizing the risk of vascular injury. Medical-grade polyurethane catheters were used for their biocompatibility, strength, and flexibility, ensuring high flow rates, patient comfort, and reduced vascular injury. Their configuration supported various therapies, including vesicant agents, parenteral nutrition, and repeated blood sampling [29].

Infection prevention was ensured through strict adherence to aseptic technique during catheter insertion, including the use of maximal sterile barriers (sterile gloves, gown, mask, cap, and large sterile drape) and meticulous skin antisepsis with a chlorhexidine-alcohol solution [30]. Hand hygiene remains a fundamental component of both insertion and maintenance procedures [31]. Moreover, ultrasound-guided insertion has become standard practice in many clinical settings, significantly reducing the risk of mechanical complications and enabling accurate catheter tip positioning—an essential factor in the prevention of thrombosis [32].

To mitigate the risk of thrombotic events, current guidelines emphasize optimal tip positioning at the cavo-atrial junction, avoidance of catheter malposition, and the selection of the smallest appropriate catheter gauge. In addition, maintenance protocols—such as regular flushing using a standardized start-stop technique, utilization of needle-free connectors and disinfection caps, and timely management of catheter occlusions—are critical for reducing both thrombotic and infectious complications. Evidence from the literature supports that the interval between dressing changes should not exceed 10 days [24,29].

### 2.4. Catheter-Related Late Complications

These devices are widely used for intermediate to long-term intravenous therapy but are often linked to complications that may affect treatment efficacy and patient safety. Ref. [33] Occlusion is among the most frequent issues, typically caused by intraluminal thrombus, fibrin sheath formation, or drug precipitates. This can partially or fully block the catheter, impairing flow and requiring in persistent cases, catheter replacement. Dislocation occurs when the device is unintentionally moved from its original position due to patient activity, poor securement, or dressing failure. Even slight displacements can affect function, raise infection risk, or cause inaccurate drug delivery [34,35]. Tip migration refers to the movement of the catheter tip due to intrathoracic pressure shifts, arm motion, or inadequate fixation. Migration into smaller veins or the atrium may trigger arrhythmias, poor drug distribution, or thrombosis [36]. Infection is a major concern, with colonization, occurring via intra- or extraluminal routes. Biofilm formation reduces antibiotic efficacy, often requiring catheter removal. Immunocompromised patients are especially at risk of bloodstream infections [14,30]. Replacement may be needed in cases of persistent occlusion, infection, or mechanical failure. Venous thrombosis is a serious and often silent complication. Risk factors include vein-to-catheter size mismatch, endothelial damage, and hypercoagulable states. Symptoms may include arm swelling, pain, or dysfunction. Thrombosis can cause long-term vein damage and limit future access, making prevention and early detection critical [29,37].

### 2.5. Patient Selection

The study population comprised adult cancer patients admitted to hospital wards who received PICC or midline insertions between March 2020 and April 2024. During this period, this cancer center was identified as a COVID-19-free hospital. In line with the national contingency decrees, all patients were screened with an oropharyngeal swab before entering the hospital and were admitted if negative and wearing an FFP2 mask.

Inclusion criteria required patients to be aged 18 or older, with a confirmed cancer diagnosis, and to have undergone at least one PICC or midline insertion during the study period. Patients whose devices were removed for reasons other than late complications, such as the completion of therapy, were excluded.

Patients were divided into two groups based on the end of the pandemic emergency in Italy. Group 1 included patients who received devices between March 2020 and March 2022 (pandemic phase), while Group 2 comprised those treated between April 2022 and April 2024 (post-pandemic phase). This allowed a clear comparison of complication rates before and after the easing of pandemic-related restrictions and the resumption of regular healthcare services.

### 2.6. Statistical Analysis

Statistical analyses were conducted using descriptive and inferential methods to evaluate the temporal distribution, participant characteristics, catheter characteristics, and adverse events within the study cohort. Continuous variables were assessed for normality using the Shapiro-Wilk test and visual inspection of the residuals. For non-normally distributed data, median, and interquartile range (IQR) were used to summarize central tendency and dispersion. Categorical data were expressed as counts (*n*) and percentages (%). For categorical data, the Chi-squared (χ2) test was used to assess associations. When expected frequencies were too low for the Chi-squared test, Fisher’s exact test was applied. In cases where Fisher’s test was computationally infeasible, *p*-values were estimated using the Monte Carlo simulation method. For continuous variables, the Kruskal–Wallis ANOVA was utilized to detect differences between groups, as these variables were not normally distributed. When the Kruskal–Wallis test identified significant differences, pairwise post-hoc comparisons were performed using Dunn’s test with Bonferroni correction to adjust for multiple comparisons. A significance threshold of *p* < 0.05 was applied across all analyses. Statistical analyses were conducted using R 4.3.3 [38].

## 3. Results

### 3.1. Sociodemographic and Clinical Patients’ Characteristics

The patients included in the study who had their catheter removed due to late complications (*n* = 550) were predominantly female (*n* = 314, 57.1%), mostly admitted to oncology wards (*n* = 227, 41.3%), and primarily diagnosed with leukemia or lymphoma (*n* = 260, 47.6%). Metastatic disease was observed in 223 patients (40.5%) (Table 1).

### 3.2. Vascular Access Devices

During the study period, a total of 4104 new catheter placements and 2291 removals were documented. Among these removals, 550 (24%) were due to late complications. Temporal distribution of patients with catheters removed due to late complications across the study period shows the highest frequency in 2021, with 153 cases (27.8%). This was followed by 2020, which recorded 130 cases (23.6%). There was a slight decline in subsequent years: 121 cases in 2022 (22%) and 124 cases in 2023 (22.5%). The year 2024 exhibited a marked decrease, with only 21 cases reported (3.8% of the cohort).

Most patients (*n* = 491, 89.3%) received peripherally inserted central catheters (PICC) while Midline catheters were used in 10.7% (*n* = 59). The median duration of vascular access placement was 126.5 days (IQR: 196), and the median duration of medication administration was 7 days (IQR: 0). Vascular access characteristics are detailed in Table 2.

### 3.3. Duration of Medication

The Kruskal–Wallis test evaluating whether significant differences exist in the distribution of the duration of medication across several years considered found chi-square χ2(4) = 36.3863 (*p* < 0.0001). To further explore these differences, a post-hoc Dunn test with Bonferroni adjustment for multiple comparisons was performed, demonstrating significant differences between several group pairs (Table 2). Specifically, the comparison between 2020 and 2021, as well as 2020 and 2022, revealed significant differences, with adjusted *p* = 0.0032 and *p* = 0.0091, respectively. Similarly, the comparison between 2020 and 2023 showed highly significant differences (*p* < 0.0001). In contrast, no significant differences were observed between 2021 and 2022 (*p* = 1.000) and between 2021 and 2024 (*p* = 0.504). No significant differences were found in the number of days with the venous device across the four years, indicating that the durations were consistent over time (χ2(4) = 1.699, *p* = 0.790).

### 3.4. Adverse Events

The study identified a significantly higher frequency of late complications during the pandemic period compared to the post-pandemic period. Of the 550 late complications recorded (see Table 3), 404 occurred during the pandemic phase, while 146 occurred in the post-pandemic phase (χ2 = 240.18, *p* < 0.001).

The types of complications varied, with infections being the most common. Suspected infections accounted for a significant proportion of complications, with a higher incidence during the pandemic (χ2 = 318.20, *p* < 0.001). Multiple comparisons between the five years are shown in Table 2.

### 3.5. Stratification by Type of Malignancy

Given the clinical relevance of different malignancy types—for instance, the contrast between immunocompromised patients with hematological cancers and those with solid tumors such as gastrointestinal malignancies—we conducted a stratified analysis to assess whether the distribution of cancer types varied over the study years. This analysis revealed a statistically significant shift in the distribution of malignancy types across time (*p* = 0.0055). Hematological malignancies (leukemias and lymphomas) constituted the predominant group in 2020 and 2021, representing 50.8% and 49.7% of cases, respectively. Their proportion steadily declined in subsequent years: 39.4% in 2022, 31.0% in 2023, and only 18.2% in 2024. In contrast, breast cancer cases showed an upward trend, increasing from 13.1% in 2020 to a peak of 20.1% in both 2022 and 2023, before slightly decreasing to 11.4% in 2024. Digestive system tumors remained relatively stable throughout the study period, with proportions ranging from 19.2% to 21.7%. Respiratory tract malignancies, though infrequent in the early years (2.3% in 2020), became more prevalent in 2022 and 2023 (5.6% and 6.5%, respectively).

To explore whether these changes in tumor type distribution had a potential impact on key clinical outcomes, we performed further stratified analyses. Although the overall Fisher’s exact tests indicated statistically significant differences in the distribution of complications, positive blood cultures, and catheter occlusion tests across tumor types (all *p* < 0.001), post-hoc pairwise comparisons adjusted using the Bonferroni method did not identify any statistically significant differences between individual tumor types. These results suggest that, despite overall heterogeneity, no specific malignancy was disproportionately associated with worse outcomes. Therefore, the observed temporal variation in cancer type distribution is unlikely to have introduced meaningful bias into the study’s main clinical findings.

In addition, Dunn’s post-hoc test (with Bonferroni correction) was employed to assess differences in catheter dwell time between malignancy types, following a significant Kruskal–Wallis test result (*p* = 0.026). While no pairwise comparison reached statistical significance after correction, two comparisons approached the threshold: hematological versus gastrointestinal tumors (adjusted *p* = 0.075) and respiratory versus breast cancers (adjusted *p* = 0.027). These trends, although not definitive, may point to clinically meaningful patterns deserving further exploration in larger or more targeted cohorts.

## 4. Discussion

The COVID-19 pandemic significantly disrupted healthcare systems worldwide, profoundly affecting the management of vascular access devices in cancer patients. Delays in cancer diagnosis, interruptions in chemotherapy schedules, and deferral of routine oncological evaluations became widespread during this period, with significant implications for patient outcomes [39,40]. These disruptions were largely due to the reallocation of resources, temporary closures of outpatient services, and prioritization of COVID-19 care over elective or chronic conditions.

Our retrospective study revealed a marked increase in late complications such as suspected infections, dislodgements, and device replacements, during the pandemic compared to the post-pandemic period. This finding underscores the heightened vulnerability of cancer patients amidst healthcare disruptions [41]. Similar studies have reported increased catheter-related complications during the pandemic, attributing them to strained healthcare resources and altered care protocols [42].

The disruption of outpatient services and the reallocation of healthcare resources towards managing COVID-19 cases likely contributed to increased complication rates observed during the pandemic. Additionally, limited availability of trained nursing personnel and reduced opportunities for regular device surveillance and maintenance were probable contributing factors [20,21]. Lack of continuous monitoring increases the risk of complications such as infections and mechanical failures, both of which were prominent in the study cohort. Notably, infections accounted for over a third of late complications, with suspected infections significantly higher during the pandemic [43]. Hospitals experienced systemic overloads, leading to reduced access to skilled vascular access teams and delays in routine PICC maintenance. These findings echo studies that reported elevated infection rates in hospitalized patients during the COVID-19 period, attributable to compromised care environments and altered infection control protocols [6,42,44].

Our study’s demographic data align with existing literature, with a mean patient age of 60.5 years and a predominance of female patients. The high prevalence of hematological malignancies, including lymphoma and leukemia, among our cohort highlights the increased susceptibility of these patients to vascular device complications due to underlying immunosuppression and intensive chemotherapy regimens. This observation is consistent with previous research indicating that patients with hematological malignancies are at higher risk for catheter-related complications [45,46,47].

The type of catheter used also influenced complication rates. PICCs constituted the majority of devices inserted, consistent with their widespread use in cancer care due to their ease of insertion and versatility in long-term treatment [3,48]. However, the extended dwell times of these devices, coupled with restricted follow-up during the pandemic, likely exacerbated risks. Previous studies have demonstrated a direct relationship between the duration of catheter use and the incidence of complications, particularly infections and thrombosis [24]. In contrast, some studies have suggested that midline catheters may be associated with a significantly higher rate of total complications compared to PICCs (relative risk= 1.95, 95% confidence interval= 1.23–3.08, *p*= 0.005, I2 = 0%) [49,50]. This discrepancy may be due to differences in study populations, catheter management protocols, or healthcare settings.

Another important but rarely discussed element is the role of healthcare worker burnout. During the pandemic, frontline staff experienced intense psychological and physical fatigue, with increased workload, long shifts, and resource shortages [51]. These conditions may have impaired procedural accuracy during catheter insertions and reduced adherence to maintenance protocols, potentially contributing to the observed increase in PICC-related complications [52].

Our findings provide compelling evidence for the need to strengthen vascular device management protocols during health emergencies. Establishing robust systems for remote monitoring and telehealth consultations could mitigate the impact of restricted in-person care. The integration of wearable technologies to monitor device function and detect early signs of complications may further enhance patient safety [53]. Moreover, expanding the role of community-based care and training non-specialized personnel to provide basic device maintenance during crises could bridge care gaps. These strategies align with recommendations from global health authorities advocating for resilience in healthcare delivery systems [54].

Finally, PICC-related complications, particularly infections and dislodgements, may lead to treatment delays, increased hospitalizations, and even treatment abandonment in oncologic patients. These outcomes can directly compromise cancer prognosis [55,56]. A more thorough understanding of long-term consequences is essential when evaluating the true burden of vascular access complications during crisis situations.

Another critical finding is the significant reduction in complication rates in the post-pandemic period. This improvement reflects the resumption of regular healthcare services, including specialized vascular device care. Enhanced infection control measures implemented post-pandemic, informed by the lessons learned during the crisis, likely contributed to this positive trend. These findings emphasize the importance of continuous quality improvement in healthcare practices to prevent future disruptions and ensure optimal patient outcomes [57,58,59].

### Limitations and Strengths

This study has several limitations. As a single-center retrospective review, its findings may not be generalizable to other settings. Variations in healthcare infrastructure, pandemic responses, and patient demographics could affect outcomes. Additionally, reliance on retrospective data may lead to incomplete or inaccurate documentation. Future research should employ multicenter designs and prospective methodologies for a broader and more reliable understanding of vascular device management in cancer care.

A potential limitation of this study is the use of calendar-year groupings to define the pandemic and post-pandemic periods, which may not fully reflect the nuanced and evolving nature of the COVID-19 crisis. This approximation was necessary due to the retrospective nature of the data and the absence of monthly granularity in the source records.

Despite these limitations, the study has notable strengths. It provides a detailed analysis of patient records during the pandemic and post-pandemic periods, offering valuable insights into the impact of healthcare disruptions on vulnerable populations.

## 5. Conclusions

The increase in late complications from PICCs and Midlines among cancer patients during the COVID-19 pandemic highlights the unintended effects of healthcare service disruptions on patient outcomes, particularly infections. Maintaining continuity in cancer care and vascular device management is vital.

The study underscores the need for robust contingency plans to sustain essential services like catheter maintenance during crises. Alternative care models and home-based support can mitigate risks from reduced in-person interactions. Educating on vascular device self-management and strengthening infection prevention training for healthcare professionals are crucial. Looking ahead, healthcare systems must implement innovative strategies to maintain high-quality care during crises. Training non-specialized personnel in basic device maintenance and developing robust contingency plans for emergency healthcare delivery can strengthen resilience. Future multicenter studies are recommended to confirm these findings across diverse healthcare settings, patient populations, and institutional practices.

## Figures and Tables

**Table 1:** Characteristics of patients (*n* = 550) and devices (PICCs and Midlines).

Variables	Value
Gender	
Female	314 (57.1%)
Male	236 (42.9%)
Age M [IQR]	63 years [17.75]
Education	
Middle school	261 (47.5%)
High school	157 (28.5%)
Bachelor	40 (7.3%)
Hospital ward	
Oncology	227 (41.3%)
Hematology	174 (31.7%)
Transplantation and Bone-Marrow Oncology	71 (12.9%)
Other	77 (14.1%)
Cancer diagnosis	
Leukemia/Lymphoma	260 (47.6%)
Digestive system cancers	121 (22.2%)
Breast cancer	81 (14.8%)
Head and neck cancers	26 (4.7%)
Metastatic disease	
Present	223 (40.5%)
Absent	327 (59,5%)
Home care §	
No	336 (62.1%)
Yes	205 (37.8%)
Indication for device insertion	
Chemotherapy	518 (94.1%)
DIVA	31 (5.58%)
Parenteral Nutrition	1 (0.2%)
In-Hospital insertion	
No	49 (8.9%)
Yes	501 (91.1%)
Device caliber	
4 French	516 (93.8%)
5 French	34 (6.2%)
Number of lumens	
Mono-lumen	524 (95.3%)
Bi-lumen	26 (4.7%)
Vein	
Basilic	353 (64.2%)
Brachial	189 (34.4%)
Cephalic	8 (1.4%)
Arm	
Left	193 (35.1%)
Right	357 (64.9%)

Legend: § = care provided at home by nurses of the Cancer Center; DIVA = Difficult Intravenous Access; IQR= Interquartile range.

**Table 2:** Multi-Year Comparisons and Post-hoc Comparisons of Medication Duration.

Variable	2020	2021	2022	2023	2024	*p*-Value
Sex						
Male	59 (45.4%)	74 (48.4%)	56 (46.3%)	39 (31.5%)	7 (33.3%)	0.035 *
Female	71 (54.6%)	79 (51.6%)	65 (53.7%)	85 (68.6%)	14 (66.7%)	
Educational Level						
First School	26 (20.2%)	20 (13.1%)	20 (16.5%)	22 (17.7%)	3 (14.3%)	0.663
Middle school	55 (42.6%)	73 (47.7%)	60 (49.6%)	63 (50.8%)	9 (42.9%)	
High school	38 (29.5%)	49 (32.0%)	29 (23.9%)	32 (25.8%)	9 (42.8%)	
Bachelor’s Degree	10 (7.7%)	11 (7.2%)	12 (9.9%)	7 (5.6%)	0 (0.0%)	
Home care *n* (%)						
No	70 (53.8%)	58 (38.4%)	79 (65.3%)	108 (92.3%)	20 (95.2%)	<0.0001 ***
Yes	60 (46.1%)	93 (61.6%)	42 (34.7%)	9 (7.7%)	1 (4.8%)	
Vascular Access *n* (%)						
Midline	15 (11.5%)	20 (13.0%)	15 (12.4%)	6 (4.8%)	3 (14.3%)	0.125
PICC	115 (88.5%)	133 (86.9%)	106 (87.6%)	118 (95.2%)	18 (85.7%)	
Indication for insertion (%)						
Chemotherapy	128 (98.5%)	149 (97.4%)	115 (95.0%)	107 (86.3%)	18 (85.7%)	0.003 **
DIVA	2 (1.5%)	4 (2.6%)	6 (4.9%)	16 (12.9%)	3 (14.3%)	
Parenteral Nutrition	0 (0.0%)	0 (0.0%)	0 (0.0%)	1 (0.81%)	0 (0.0%)	
In-Hospital insertion *n* (%)						
No	18 (13.8%)	10 (6.5%)	9 (7.4%)	12 (9.7%)	0 (0.0%)	0.154
Yes	112 (86.1%)	143 (93.5%)	112 (92.6%)	112 (90.3%)	21 (100.0%)	
Caliber *n* (%)						
4 French	127 (97.7%)	148 (96.7%)	109 (90.1%)	113 (91.1%)	18 (85.2%)	0.008 **
5 French	3 (2.3%)	5 (3.3%)	12 (9.9%)	11 (8.9%)	3 (14.2%)	
Lumen *n* (%)						
Mono-lumen	128 (98.5%)	147 (96.0%)	108 (89.2%)	121 (97.6%)	19 (90.5%)	0.003 **
Bi-lumen	2 (1.5%)	6 (4.0%)	13 (10.7%)	3 (2.4%)	2 (9.2%)	
Vein *n* (%)						
Basilic	85 (65.4%)	107 (70.0%)	68 (56.2%)	77 (62.1%)	15 (71.4%)	0.125
Brachial	42 (32.3%)	44 (28.7%)	51 (42.1%)	47 (37.9%)	5 (23.8%)	
Cephalic	3 (2.3%)	2 (1.3%)	2 (1.6%)	0 (0.0%)	1 (4.7%)	
Arm *n* (%)						
Left	36 (27.7%)	51 (33.3%)	49 (40.5%)	44 (35.5%)	13 (61.9%)	0.013 *
Right	94 (72.3%)	102 (66.6%)	72 (59.5%)	80 (64.5%)	8 (38.1%)	
Complications *n* (%)						
Dislocation	29 (22.3%)	33 (21.6%)	18 (14.8%)	40 (32.3%)	5 (23.8%)	<0.0001 ***
Mechanical injury	7 (5.4%)	3 (2.0%)	3 (2.4%)	1 (0.8%)	0 (0.0%)	
Tip migration	3 (2.3%)	2 (1.3%)	0 (0.0%)	0 (0.0%)	0 (0.0%)	
Occlusion	25 (19.2%)	40 (26.1%)	19 (15.7%)	21 (16.9%)	7 (33.3%)	
Suspected infection	55 (42.3%)	58 (37.9%)	41 (33.8%)	26 (20.9%)	5 (23.8%)	
Replacement with another device	9 (6.9%)	13 (8.5%)	34 (28.1%)	32 (25.8%)	4 (19.0%)	
Venous thrombosis	2 (1.5%)	4 (2.6%)	6 (4.9%)	4 (3.2%)	0 (0.0%)	
Blood culture *n* (%)						
Positive	36 (31.0%)	39 (25.5%)	24 (20.5%)	28 (22.7%)	6 (30.0%)	<0.0001 ***
Negative	20 (17.2%)	22 (14.4%)	13 (11.1%)	9 (7.3%)	2 (10.0%)	
Not performed	60 (51.7%)	92 (60.1%)	80 (68.4%)	86 (69.9%)	12 (60.0%)	
Infectious agent *n* (%)						
Aerobic	17 (50.0%)	15 (39.5%)	7 (36.8%)	5 (35.7%)	0 (NA)	0.013 *
Anaerobic	1 (2.9%)	3 (7.9%)	4 (21.0%)	0 (0.0%)	0 (NA)	
Aerobic and Anaerobic	16 (47.0%)	20 (52.6%)	8 (42.1%)	9 (64.3%)	0 (NA)	
Tip culture *n* (%)						
Negative	16 (13.9%)	11 (7.3%)	0 (0.0%)	2 (1.7%)	3 (15.0%)	<0.0001 ***
Positive	32 (27.8%)	35 (23.2%)	38 (31.9%)	23 (19.2%)	4 (20.0%)	
Not performed	67 (58.3%)	105 (69.5%)	81 (68.0%)	95 (79.2%)	13 (65.0%)	
Occlusion test *n* (%)						
Negative	18 (16.4%)	26 (17.2%)	15 (12.5%)	6 (4.9%)	5 (25.0%)	<0.0001 ***
Positive	87 (79.1%)	124 (82.1%)	101 (84.2%)	38 (30.9%)	1 (5.0%)	
Not performed	5 (4.5%)	1 (0.6%)	4 (3.3%)	79 (64.2%)	14 (70.0%)	
Post-hoc Comparisons of Medication Duration						Adjusted *p*-value
2020–2021		3.4165				0.003 ***
2020–2022	3.1173		3.1173			0.009 ***
2021–2022			−113			1.000
2020–2023	5.723			5.723		0.000 ***
2021–2023		2.5726		2.5726		0.051
2022–2023				2.5402		0.055
2020–2024	3.3172				3.3172	0.004 ***
2021–2024		1.6012			1.6012	0.546
2022–2024			1.6344		1.6344	0.511
2023–2024					0.2617	1.000

Based on a Monte Carlo simulation of *n* = 2000 replications; Significant at: * = 0.05; ** = 0.01; *** = 0.001; DIVA = Difficult Intravenous Access.

**Table 3:** Adverse Events.

Complications *n* (%)	
Occlusion	122 (20.4%)
Dislocation	125 (22.7%)
Mechanical injury	14 (2.5%)
Tip migration	5 (0.9%)
Suspected infection	185 (33.6%)
Replacement with another device	93 (16.9%)
Venous thrombosis	16 (2.9%)
Blood culture *n* (%)	
Positive	133 (25.1%)
Negative	66 (12.4%)
Not performed	331 (62.4%)
Infectious agent *n* (%)	
Aerobic	44 (41.9%)
Anaerobic	8 (7.6%)
Aerobic and Anaerobic	53 (50.5%)
Positive tip culture *n* (%)	
No	32 (6.1%)
Yes	132 (25.2%)
Not performed	359 (68.6%)
Occlusion test *n* (%)	
Negative	70 (13.3%)
Positive	351 (66.8%)
Not performed	104 (19.8%)

## Data Availability

Data are available on request.
